# Assessing trends in non-coverage bias in mobile phone surveys for estimating insecticide-treated net coverage: a cross-sectional analysis in Tanzania, 2007–2017

**DOI:** 10.1136/bmjph-2024-001379

**Published:** 2025-03-04

**Authors:** Matt Worges, Ruth A Ashton, Janna Wisniewski, Paul Hutchinson, Hannah Koenker, Tory Taylor, Hannah Metcalfe, Ester Elisaria, Mponeja P Gitanya, Charles Dismas Mwalimu, Frank Chacky, Joshua O Yukich

**Affiliations:** 1 Tropical Health, New Orleans, Louisiana, USA; 2 Tulane University School of Public Health and Tropical Medicine, New Orleans, Louisiana, USA; 3 Tropical Health LLP, Baltimore, Maryland, USA; 4 The University of North Carolina, Carolina Population Center, Chapel Hill, North Carolina, USA; 5 Viamo, Dar es Salaam, Tanzania, United Republic of; 6 Department of Impact Evaluation, Ifakara Health Institute, Ifakara, Dar es Salaam, Tanzania, United Republic of; 7 National Malaria Control Program, Ministry of Health, Dodoma, Tanzania, United Republic of

**Keywords:** Public Health, Population Surveillance, Cross-Sectional Studies, trends

## Abstract

**Introduction:**

Monitoring insecticide-treated net (ITN) coverage and use generally relies on household surveys which occur on a relatively infrequent basis. Because indicators of coverage are used to forecast the need for ITNs and aid in planning ITN distribution campaigns, higher frequency monitoring could be helpful to guide programme strategies. The use of mobile phone-based survey (MPS) strategies in low-income and middle-income countries has emerged as a rapid and comparatively inexpensive complement to large-scale population-based household surveys, considering the dramatic growth trend of mobile phone ownership.

**Methods:**

The potential for non-coverage bias in the calculation of ITN coverage estimates from MPSs was assessed through the use of five consecutive Tanzania-specific Demographic and Health Surveys (DHS). Primary comparisons were made between all households included in the data sets (the reference standard) and mobile phone-owning households (the comparator). Deviations in ITN coverage estimates between the reference standard and mobile phone-owning households were used as a proxy for assessing potential non-coverage bias, with estimates calculated using a bootstrap method.

**Results:**

By the 2017 DHS, regional measures of non-coverage bias for ITN coverage indicators rarely exceeded a ±3 percentage point difference when comparing mobile phone-owning households to the overall sample. However, larger differences were observed when comparing mobile phone-owning households to non-mobile phone-owning households, particularly in periods without recent mass ITN distributions.

**Conclusion:**

Results suggest that MPSs can reliably estimate ITN coverage at the population level when both ITN coverage and mobile phone ownership are high. However, as ITN coverage declines, the gap between phone-owning and non-phone-owning households widens, indicating potential non-coverage bias and underscoring the need for caution in interpreting MPS data under such conditions.

**WHAT IS ALREADY KNOWN ON THIS TOPIC**

- Evidence from recent studies conducted in low-income and middle-income countries shows that mobile phone surveys (MPSs) tend to oversample male, urban, younger and better-educated respondents suggesting that non-coverage bias remains an issue.

**WHAT THIS STUDY ADDS**

- This study focused on assessing the potential for non-coverage bias by comparing standard insecticide-treated net (ITN) coverage indicators estimated from a sample of mobile phone-owning households compared with a sample of all households in Tanzania. This comparison provides some insight into whether excluding non-phone-owning households in an MPS conducted in Tanzania would lead to systematic differences in estimates of ITN coverage indicators.

**HOW THIS STUDY MIGHT AFFECT RESEARCH, PRACTICE OR POLICY**

- Carefully designed MPS, perhaps with the use of weighting for analysis, could serve as an interim or supplemental data collection method capable of providing programme administrators with more frequent monitoring of specific ITN indicators.

## Introduction

 Since the early 2000s, Tanzania has expanded malaria prevention and control efforts, including improvements in case management, distribution of insecticide-treated nets (ITNs) and the use of indoor residual spraying. These strategies have driven a significant decline in malaria prevalence among children. However, recent data from the 2022 Tanzania Demographic and Health Survey and Malaria Indicator Survey (DHS-MIS) suggest that this decline may be levelling off, with prevalence among children aged 6–59 months dropping from 14% in 2015–2016 to 7.5% in 2017 and to 8% in 2022.^1^,^3^ There is strong regional variation in malaria prevalence ranging from 0% to 23.4% in 2022.^3^

The Tanzanian government has maintained a longstanding focus on malaria control through extensive support for ITN distribution. The National ITN programme, initiated in 2000, marked a concerted effort to scale up ITN coverage and use.^4^ Other key initiatives included the National Voucher Scheme (2004–2014), which provided nets at a subsidised cost through private commercial shops for pregnant women and children attending routine antenatal and immunisation clinic visits, and a health facility-based distribution system (2014–2016, restarted in 2018).^5^,^7^ By 2017, Tanzania had conducted three mass ITN distribution campaigns, including the 2009–2010 Under-Five Catch-Up Campaign and two Universal Coverage Campaigns (2010–2011, 2015–2017).^8 9^ Beginning in 2013, the School Net Programme was developed as an additional ‘keep-up’ distribution strategy to maintain ITN coverage levels.^10^ The various ITN distribution channels used in Tanzania are proven cost-effective strategies for malaria control in low-income and middle-income countries (LMICs) and the use of ITNs confers both personal-level and community-level protection when coverage targets are achieved.^11^,^15^

In order to track population-level indicators of ITN ownership, access and use, periodic postdistribution campaign assessments are carried out through expanded surveillance efforts.^916^,^19^ The measurement of ITN coverage generally relies on household surveys which are expensive and time-consuming^20^ and occur relatively infrequently. Standard DHSs, which typically include an ITN submodule for epidemiologically relevant settings, are nationally representative household surveys carried out, on average, every 5 years at an estimated cost of US$1.6 million per survey (based on a sample of 30 LMIC).^21^ Between rounds of the Standard DHS surveys, key indicators related to ITNs can still be monitored through Interim DHS surveys. The Tanzanian government has conducted six Standard or Interim DHS surveys between 2004 and 2017, on average every 2.5 years.^12 22^,^25^ Among many other indicators, these surveys measure ITN ownership, access and use, which are used to forecast the need for ITNs and aid in planning ITN distribution campaigns. However, higher frequency monitoring could be helpful to guide programme strategies, particularly for countries using combinations of mass campaigns, targeted campaigns and continuous distribution, due to the need to annually check and adjust continuous ITN distribution to maintain high levels of ITN access. Additionally, field evidence increasingly demonstrates that ITN functional survival is shorter than WHO assumptions.^26^,^31^

The use of mobile phone-based survey (MPS) strategies in LMIC has emerged as a rapid and comparatively inexpensive complement to large-scale population-based household surveys considering the dramatic growth trend of mobile phone ownership in such countries.^32^,^38^ Indeed, a cost study conducted in Bangladesh, Colombia and Uganda for mobile phone health surveys using interactive voice response for assessing risk factors of non-communicable diseases estimated fixed costs ranging between US$47 000 and US$74 000 (2017 USD) and variable costs between US$32 000 and US$129 000 (2017 USD).^37^ An 18-question random digit dial survey conducted in Ghana calculated a cost of US$4.95 (2017 USD) per complete survey, which totalled just under US$47 000.^39^ The authors of this paper have also conducted a random digit dial interactive voice response MPS in Tanzania with no participant incentives at the cost of US$11.39 (2017 USD) per complete interview for a total cost of just over US$22 000 (manuscript under preparation).

In 2017, about 75%–80% of adult respondents from surveyed households in Tanzania reported owning a mobile phone, where ownership was more common among men, individuals aged 30–49 years, those with at least a secondary level of education and people living in households with an income level above the country median.^2 40^ Handset and/or SIM cost was cited as the most common barrier to mobile phone ownership in Tanzania among those individuals who did not already own one.^41^ In 2012, approximately 3.4 million Tanzanians (about 7% of the population at the time), mainly from rural locales, were living outside of mobile phone coverage areas.^42^ In 2017, there was an estimated 23.7 million unique subscribers to mobile network operators translating to 42% penetration (ie, the number of SIM cards in use across the entire population)—an increase from 10.4 million subscribers in 2008.^43 44^ An additional six million new subscribers were expected by 2025.^45^ Tanzania, alongside the Democratic Republic of the Congo, Ethiopia and Nigeria, was estimated to collectively account for nearly half of all new mobile phone subscribers in Africa by 2020.^43^ It is important to note, however, that penetration rates tend to overestimate mobile phone coverage of the population.^46^

As in all surveys, non-coverage and non-response error are potential issues when conducting MPS in LMIC. Non-coverage error occurs when individuals in the population of interest have no way of being selected to participate in the survey (eg, they do not own or do not have access to a telephone in the context of an MPS). Non-response error occurs when a selected individual does not participate (or incompletely participates) in the survey. As mobile phone penetration rapidly expanded across certain LMICs, non-coverage error presumably declined as a larger proportion of the population gained access.^47 48^ However, with the growth rate in new ownership slowing, as is the case for Tanzania, non-coverage error will continue to be an issue for population segments that remain without access. Additionally, evidence from recent studies shows that MPSs tend to oversample male, urban, younger and better-educated respondents—all of whom are generally more likely to own mobile phones in LMIC settings—suggesting that non-coverage bias is a potential issue.^38^,^4046 49 50^ MPSs in Africa tend to under-represent women, but it is not always clear whether this under-representation is due to non-coverage error—where women are less likely to have access to mobile phones—or non-response bias, where women, despite having access, may be less likely to participate in the surveys due to the inconvenience given demands associated with childcare and household management.^39 51^ This paper aims to assess the potential for non-coverage bias by comparing Roll Back Malaria Monitoring and Evaluation Reference Group (RBM-MERG) ITN coverage indicators estimated from a sample of mobile phone-owning households compared with a sample of all households in Tanzania. This analysis provides insights into whether ITN coverage estimates from an MPS would be systematically biased by excluding non-phone-owning households.

## Methods

The data sources used for this study are from five consecutive Tanzania-specific, nationally representative Standard and Interim DHS surveys. Specifically exploited were the 2007–2008 HIV/AIDS Indicator Survey (AIS) and MIS, the 2010 DHS, the 2011–2012 AIS-MIS, the 2015–2016 DHS and the 2017 MIS. Data sets and descriptions of the methods used to implement the DHS surveys can be found on the DHS website (https://dhsprogram.com). A question on household-level mobile phone ownership was first asked in the 2007–2008 survey and has since been asked in subsequent surveys with slight variation. In the 2007–2008 and the 2010 surveys, the question was the same and read “Does your household have a mobile telephone?” while in both the 2015–2016 and 2017 surveys, the question read “Does any member of this household own a mobile phone?”. The 2011–2012 survey had a question that read “Does your household have a mobile telephone in working condition?”.

### Statistical methods

The potential for non-coverage bias was explicitly assessed through comparisons of all households included in the data sets (the reference standard) and mobile phone-owning households only (the comparator). Deviations in ITN coverage estimates between the reference standard and the comparator provide estimates of potential non-coverage bias, indicating how the exclusion of non-phone-owning households could impact survey results. An implicit assumption is that mobile phone-owning households included in the DHS data sets are reflective of those households that would be reached by and respond to an MPS under real-world conditions. Responses for calculating ITN indicators were assessed at the household level, without accounting for potential differences in reporting between men and women regarding asset ownership and household size.

Statistical analysis was carried out using R V.4.0.1.^52^ Conditional density plots were constructed for mobile phone ownership status and characterised by a harmonised wealth index,^53^ location of residence and survey year. Multivariable cluster-adjusted models were fit to the 2017 MIS data with household-level mobile phone and ITN ownership as separate outcomes. For purposes of regional comparisons over time, administratively split regions were rejoined to their 2008 administrative boundaries where possible. Geita, Shinyanga, Mwanza, Kagera and Simiyu were grouped into a single entity which represents five of the six regions constituting the Lake Zone.

To assess the magnitude of potential non-coverage bias, ITN ownership and access indicators were estimated using a bootstrap method where data were resampled 10 000 times with replacement from each region of each DHS survey. This process was conducted for all households in each survey and then repeated for both scenarios where a mobile phone was and was not reported in the household. Mean percentage point differences by indicator, region and DHS survey were calculated between the overall and mobile phone-owning samples as well as between the mobile phone-owning and non-owning samples to give unadjusted and unweighted crude estimations of non-coverage bias.

### Spatial analysis

Using the R-INLA package,^54^ a Bayesian inference spatial smoothing approach using Integrated Nested Laplace Approximation (INLA) was applied in order to fit geostatistical models predicting ITN ownership among all households (the reference standard) as well as households reporting mobile phone ownership for each assessed survey. Non-coverage bias was estimated as the absolute difference between these two spatial layers (ie, a third spatial layer was generated to spatially depict bias). A spatio-temporal model was also created via INLA to depict the distribution of household-level mobile phone ownership from 2008 to 2017. This model specification assumed a linear effect of time for each area and made use of guidance published by Toh *et al*.^55^ Covariate raster layers used in the INLA procedures were created for travel time to the nearest city of 40 000 or more inhabitants, the 2014 built population (ie, an index ranging from 0.00 (extremely rural) to 1.00 (extremely urban)),^56^ population contemporaneous to the year of each survey,^57^ land cover classification,^58^ population-weighted educational attainment for women of reproductive age^59 60^ and prevalence of improved housing.^61^ Each covariate raster layer was resampled to match the resolution (4.6 km by 4.6 km) and extent of the improved housing raster layer, ensuring alignment across all covariates. For continuous variables, bilinear interpolation was applied, and nearest-neighbour interpolation was used for categorical variables. The choice of resolution was guided by a balance between spatial precision and computational feasibility. While finer resolutions could offer more detailed spatial representation, they would result in prohibitively long compiling times for the INLA models.

### RBM-MERG indicators

Three standard bed net indicators recommended by the RBM-MERG were used to assess ITN coverage. These indicators are endorsed by the WHO and are used globally to measure ITN coverage and guide malaria control efforts. Taken together, they provide a comprehensive measure of ITN availability and potential population-level coverage allowing for an evaluation of coverage gaps that may occur when ITNs are insufficiently distributed or not equally accessible to all household members.

1. **Proportion of households with at least one ITN**: This indicator measures the percentage of households that own at least one ITN, providing insight into the basic availability of ITNs at the household level. While it reflects the presence of ITNs, it does not account for whether there are sufficient nets to protect all household members.
  - Numerator: Number of households surveyed with at least one ITN.
  - Denominator: Total number of households surveyed.
2. **Proportion of households with at least one ITN for every two people (full household ITN coverage**): To determine whether households are adequately equipped to protect all members, the proportion of households with enough ITNs to provide full coverage, defined as one ITN for every two people, was calculated. This indicator aligns with WHO recommendations and evaluates whether ITN distribution meets the needs of the entire household.
  - Numerator: Number of households with at least one ITN for every two people (net to person ratio ≥0.5).
  - Denominator: Total number of households surveyed.
3. **Proportion of population with access to an ITN in their household**: This population-level indicator estimates the percentage of individuals who have access to an ITN within their household, regardless of actual use. It measures potential coverage, offering a clearer view of whether all household members can access ITNs, bridging the gap between household ownership and individual-level protection.
  - Numerator: Total number of individuals who could sleep under an ITN if each ITN in the household were used by two people.
  - Denominator: Total number of individuals who spent the previous night in surveyed households.

### Patient and public involvement

Neither the public nor study participants were involved in the conceptualisation of the secondary data analyses undertaken for the work presented in this manuscript.

## Results

Response rates for the five Tanzania DHS surveys used in this analysis were between 97.6% and 99.4% with an average of about 10 000 households interviewed per nationally representative survey (full details are included in the publicly available DHS reports). All five DHS surveys included an ITN submodule, but the 2010 DHS did not include a malaria biomarker component, and the 2017 MIS did not include educational attainment for the head-of-household. Periods of data collection for each survey are depicted in online supplemental figure 1 and are positioned in relation to timeframes for the different ITN distribution events used in Tanzania.

Descriptive household-level attributes are shown in table 1. The harmonised wealth index was calculated from a combined data set including all five DHS surveys, hence the unequal distribution of households into wealth quintiles for any given survey. Across surveys, an overall decline is noted for the proportion of households in the lowest two wealth quintiles, with an overall gain in the proportion of households falling into the top three wealth quintiles. Mobile phone ownership increased by over 50 percentage points from 28.1% in 2007–2008 to 81.5% in 2017. Nearly one-quarter (22.1%) of households sampled for the 2017 MIS did not own an ITN, and 17.7% of households reported no mosquito net ownership of any kind.

**Table 1:** Household-level attributes by survey

Household attributes	TZ AIS-MIS 2007–2008	TZ DHS 2010	TZ AIS-MIS 2011–2012	TZ DHS 2015–2016	TZ MIS 2017
	N=8419	N=9568	N=9980	N=12 552	N=9322
	n (weighted %)	n (weighted %)	n (weighted %)	n (weighted %)	n (weighted %)
Mobile phone ownership					
Mobile phone in household	2594 (28.1)	4533 (46.2)	6197 (61.2)	9942 (78.0)	7572 (81.6)
No mobile phone in household	5825 (71.9)	5035 (53.8)	3783 (38.8)	2610 (22.0)	1750 (18.4)
ITN ownership					
At least one ITN in household	3931 (39.1)	6321 (64.0)	8943 (91.0)	8440 (65.6)	7223 (77.9)
No ITN in household	4488 (60.9)	3247 (36.0)	1037 (9.0)	4112 (34.4)	2099 (22.1)
Any mosquito net ownership					
At least one net in household	5193 (56.2)	7350 (75.2)	9323 (94.6)	9216 (72.5)	7552 (82.3)
No net in household	3226 (43.8)	2218 (24.8)	657 (5.4)	3336 (27.5)	1770 (17.7)
Head of household age					
18–29 years	1209 (15.6)	1290 (14.9)	1376 (14.2)	1893 (15.8)	1362 (15.3)
30–49 years	4021 (48.3)	4617 (48.8)	4767 (49.0)	5970 (47.9)	4448 (48.4)
50+ years	3189 (36.1)	3661 (36.3)	3837 (36.8)	4689 (36.2)	3512 (36.2)
Head of household sex					
Male	6398 (75.6)	7274 (75.8)	7603 (75.8)	9492 (75.5)	7004 (74.6)
Female	2021 (24.4)	2294 (24.2)	2377 (24.2)	3060 (24.5)	2318 (25.4)
De jure household size					
1–2 members	1488 (19.3)	1645 (18.6)	1738 (18.5)	2465 (20.7)	1913 (22.3)
3–4 members	2357 (29.1)	2830 (30.5)	2832 (28.4)	3689 (29.9)	2717 (30.3)
5–6 members	2279 (26.5)	2573 (26.3)	2655 (27.0)	3253 (26.1)	2357 (25.6)
7–8 members	1377 (15.1)	1515 (15.0)	1596 (15.0)	1865 (14.3)	1365 (13.5)
≥9 members	918 (10.0)	1005 (9.5)	1159 (11.1)	1280 (9.0)	970 (8.3)
Head of household education					
No education	2509 (27.2)	2540 (24.1)	2350 (22.2)	2622 (19.7)	--
Primary (incomplete)	1667 (20.1)	1859 (18.9)	1893 (18.0)	2109 (16.6)	--
Primary (complete)	3183 (44.3)	3842 (45.9)	4441 (48.6)	5474 (47.0)	--
Secondary or higher	1060 (8.4)	1327 (11.0)	1296 (11.2)	2347 (16.7)	--
Wealth quintile (from HWI)					
Lowest	2462 (31.8)	2568 (26.5)	2182 (22.5)	1704 (14.4)	1133 (10.9)
Second	1710 (20.3)	1971 (20.7)	2045 (19.7)	2490 (20.0)	1746 (17.5)
Middle	1537 (16.8)	1716 (17.7)	2065 (19.9)	2497 (19.4)	2097 (21.2)
Fourth	1570 (17.4)	1722 (17.5)	1940 (19.1)	2735 (20.0)	1979 (21.5)
Highest	1140 (13.7)	1591 (17.5)	1748 (18.9)	3126 (26.1)	2367 (28.9)
Location of residence					
Rural area	1818 (24.8)	2200 (26.1)	2244 (25.5)	3629 (32.9)	2640 (33.7)
Urban area	6601 (75.2)	7368 (73.9)	7736 (74.5)	8923 (67.1)	6682 (66.3)

AIS, AIDS Indicator Survey; DHS, Demographic and Health Survey; HWI, Harmonised Wealth Index; ITN, insecticide-treated net; MIS, Malaria Indicator Survey; TZ, Tanzania.

**Figure 1:**
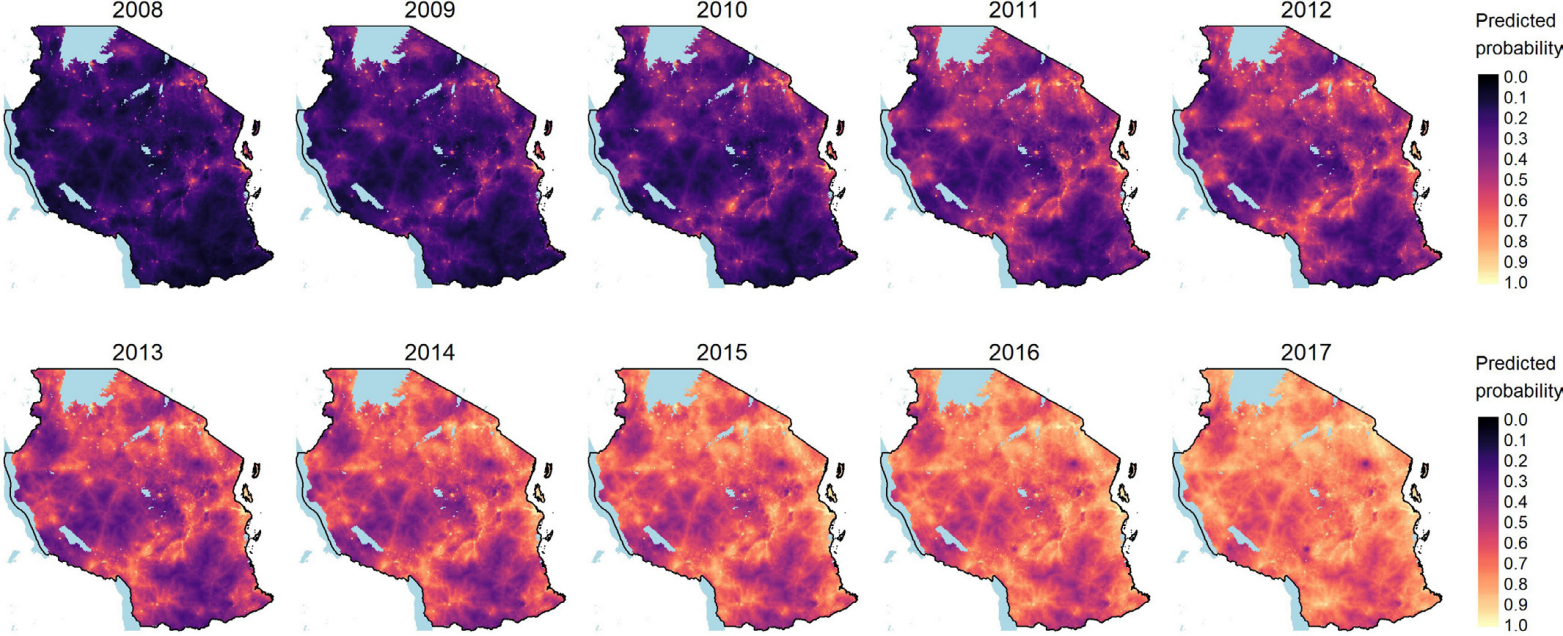
Predicted probability of household-level mobile phone ownership (2008–2017) from the final spatio-temporal model.

Conditional density plots were used to graphically depict changes in household-level mobile phone ownership over time and show large increases across the lower half of the HWI spectrum in both urban and rural settings (online supplemental figure 2). The results of the spatiotemporal model of mobile phone ownership from 2008 to 2017 are shown in figure 1. Household-level mobile phone ownership expanded rapidly, initially increasing in prevalence along major thoroughfares and eventually diffusing across the country.

In the adjusted logistic regression models (table 2), which only made use of data from the 2017 MIS, the significant predictors of mobile phone ownership included households owning at least one ITN, households headed by males, larger household size, wealthier households and households in urban areas. Mobile phone ownership was a significant predictor of ITN ownership (adjusted OR (aOR) (95% CI): 1.39 (1.16 to 1.67)) at the time of the 2017 MIS.

**Table 2:** Adjusted OR and 95% CIs of household mobile phone and ITN ownership—2017 Malaria Indicator Survey

Household-level attributes	Mobile phone ownership			ITN ownership		
	aOR	95% CI	P value	aOR	95% CI	P value
(Intercept)	0.21	0.15 to 0.30	<0.001	0.75	0.55 to 1.03	0.0780
Mobile phone ownership						
Does not own a mobile phone	--			Ref.		
Owns a mobile phone	--	--	--	1.39	1.16 to 1.67	<0.001
ITN ownership						
Does not own an ITN	Ref.			--		
Owns at least 1 ITN	1.35	1.13 to 1.62	0.0013	--	--	--
Head of household age						
18–29 years	Ref.			Ref.		
30–49 years	0.89	0.70 to 1.14	0.3611	1.40	1.13 to 1.74	0.0022
50+ years	0.61	0.47 to 0.78	<0.001	1.73	1.40 to 2.13	<0.001
Head of household sex						
Female	Ref.			Ref.		
Male	1.68	1.42 to 1.98	<0.001	0.98	0.83 to 1.16	0.8025
De jure household size						
1–2 members	Ref.			Ref.		
3–4 members	2.21	1.81 to 2.69	<0.001	2.14	1.80 to 2.53	<0.001
5–6 members	2.95	2.35 to 3.69	<0.001	2.27	1.83 to 2.81	<0.001
7–8 members	4.62	3.56 to 5.98	<0.001	2.18	1.70 to 2.79	<0.001
≥9 members	8.85	6.26 to 12.50	<0.001	1.84	1.41 to 2.41	<0.001
Wealth quintile (from HWI)						
Lowest	Ref.			Ref.		
Second	2.30	1.82 to 2.91	<0.001	1.43	1.17 to 1.74	<0.001
Middle	6.20	4.83 to 7.96	<0.001	1.37	1.08 to 1.75	0.0111
Fourth	9.92	7.38 to 13.35	<0.001	1.50	1.18 to 1.90	0.0010
Highest	36.77	26.26 to 51.48	<0.001	1.50	1.12 to 2.00	0.0066
Location of residence						
Rural area	Ref.			Ref.		
Urban area	6.98	5.67 to 8.59	<0.001	1.33	1.07 to 1.64	0.0091

aOR, adjusted OR; HWI, Harmonised Wealth Index; ITN, insecticide-treated net; Ref, reference.

**Figure 2:**
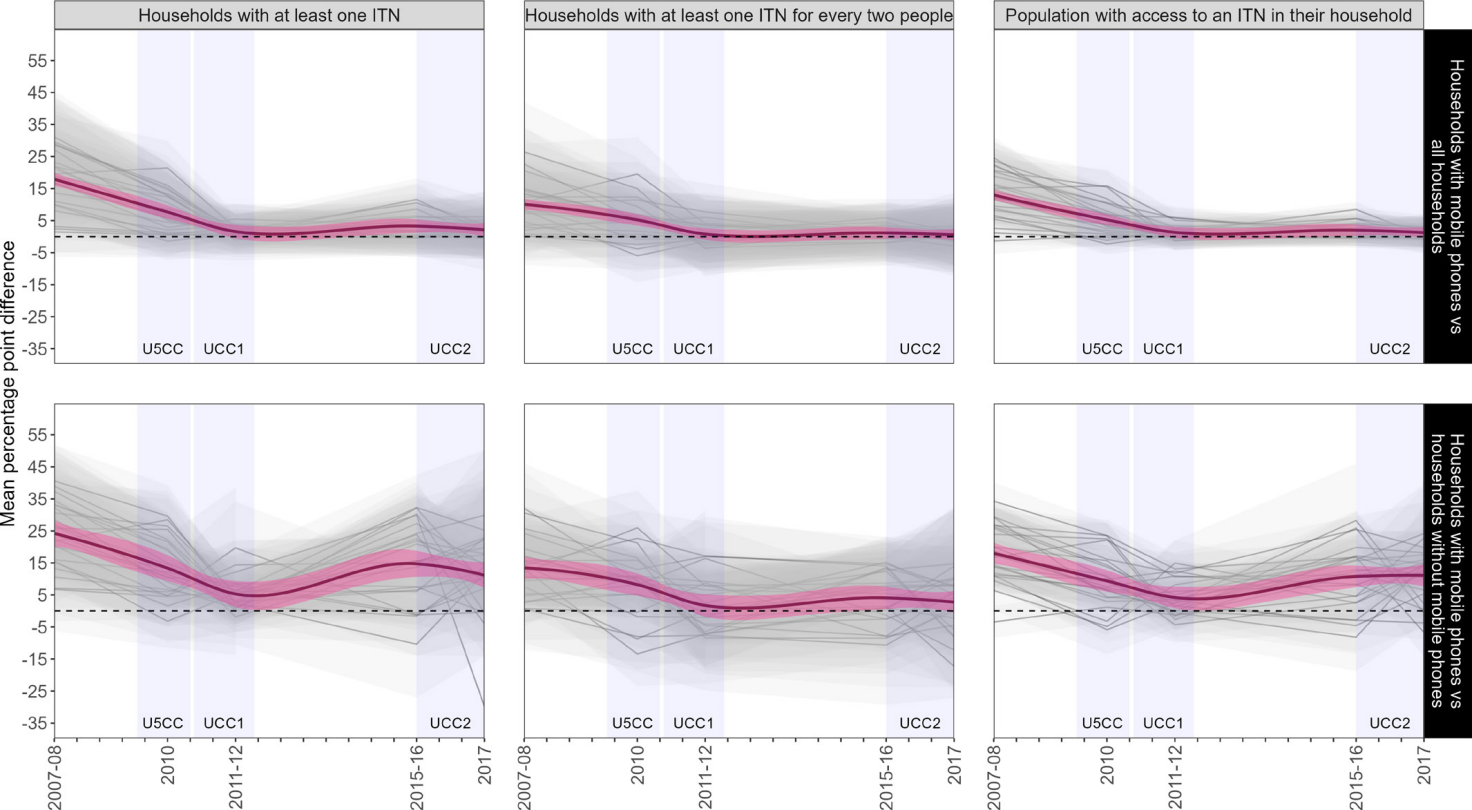
Bootstrap results for regional estimates of non-coverage bias between mobile phone-owning households and all households (upper panels) and between mobile phone-owning households and non-mobile phone-owning households (lower panels) for ownership of at least one ITN (left panels), ownership of at least one ITN per two de facto household population (centre panels), and proportion of population with access to an ITN in their household (right panels). The y-axis represents the mean difference (eg, non-coverage bias) in regional estimates between the two sample populations. The thick, darker line is a LOESS smooth line of the 24 regions (thin, lighter lines) included in the analysis. The dotted line represents zero non-coverage bias. The vertical, shaded regions show the time frame during which mass ITN distribution campaigns were undertaken. ITN, insecticide-treated net; U5CC, Under-5 Coverage Campaign; UCC1, Universal Coverage Campaign One; UCC2, Universal Coverage Campaign Two.

Bootstrap results for estimates of non-coverage bias in mobile phone-owning households against all households show a drastic decline in the mean percentage point difference between the two sample populations for the three ITN coverage indicators that were assessed (upper panels of figure 2). Declines in these indicators are noted in the presence of increasing household-level mobile phone ownership and national-scale mass ITN distribution campaigns. Nationally, the mean percentage point difference in estimates of household ownership of at least one ITN dropped from 20 at the time of the 2007–2008 AIS-MIS to 2.5 at the time of the 2017 MIS. Similar overall trends were noted for the other two ITN coverage indicators assessed. Nationally, the indicator for households with at least one ITN for every two people showed less than a ±1 percentage point swing between mobile phone-owning households and all households from the 2011–2012 survey forward. At the time of the 2017 MIS, the largest regional difference between bootstrap estimates for household ownership of at least one ITN and household ownership of at least one ITN for every two people was 6.2 and 3.1 percentage points, respectively. The largest regional difference in population access to an ITN was 4.5 percentage points.

**Figure 3:**
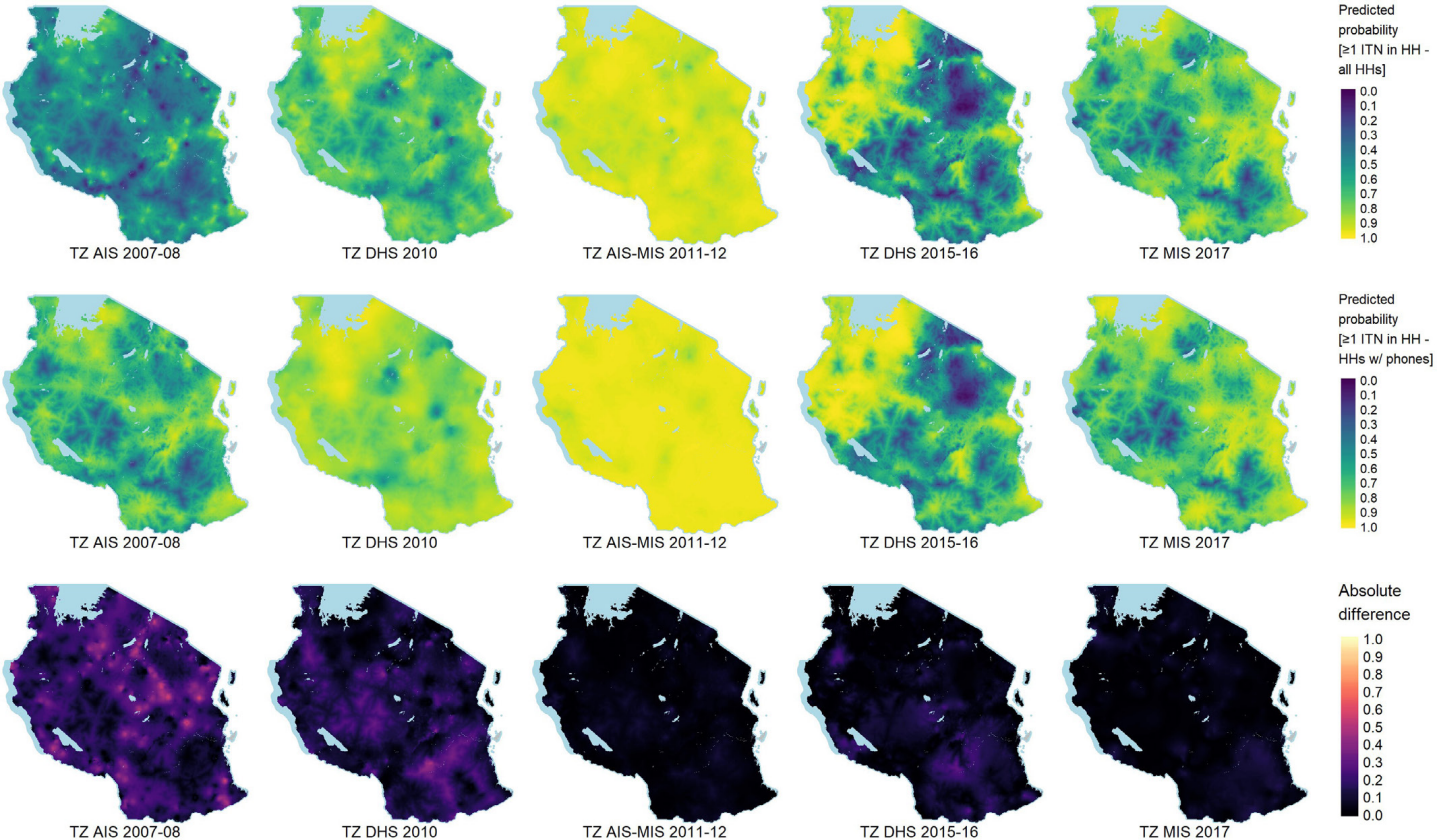
Spatial distribution of predicted ITN ownership by DHS survey using INLA. Predicted probability of household-level ownership of at least one ITN among all households (top row), predicted probability of household-level ownership of at least one ITN among mobile phone-owning households only (middle row) and absolute difference in ownership estimates of at least one ITN between all households and those with mobile phones (bottom row). DHS, Demographic and Health Survey; HH, household; INLA, Integrated Nested Laplace Approximation; ITN, insecticide-treated net; MIS, Malaria Indicator Survey; TZ, Tanzania.

In the comparison of bootstrap estimates for households with mobile phones against those without mobile phones (lower panels of figure 2), a drastic decrease in mean percentage point difference is noted over the first three survey time points. This is likely due to intensive efforts by the government to achieve universal ITN coverage between 2009 and 2011 (see ITN ownership distribution across all households in figure 3). By 2015, however, there appeared to be comparatively large differences in household ownership of at least one ITN and population access to an ITN which, despite large increases in mobile phone ownership over this time, may be due to the relatively long period during which no mass ITN distributions occurred. The regional differences in 2017 measures of one ITN per two people between households with and without mobile phones ranged from −17.3 to 14.7 percentage points, with a general trend favouring higher ITN access in households with phones. The overall average difference for this indicator by the time of the 2017 MIS was only 2.7 percentage points, although significant regional variability exists, including six regions where non-phone-owning households had greater access. Point estimates from the bootstrap results for national and regional estimates between all households, those owning a mobile phone and those without a mobile phone for each ITN coverage indicator and each assessed DHS survey are included in onlinesupplemental materials 3^7^.

The results of the INLA spatial interpolation for ITN ownership by all households and only those reporting mobile phone ownership are shown in the top and middle rows of figure 3, respectively. Ownership of at least one ITN is partially a function of survey proximity in time to mass ITN distribution campaigns. For instance, the 2011–2012 AIS-MIS was carried out following two multiyear mass ITN campaign efforts, the results of which led to near universal coverage with 90%–100% of households owning at least one ITN. Additionally, the 2015–2016 DHS survey was implemented during the second Universal ITN Coverage Campaign and likely sampled a subset of households in the northwestern area of the country that had received their nets before other areas of the country. A spatially explicit estimate of non-coverage bias in ITN ownership is depicted in the bottom row of images shown in figure 3 and is calculated as the absolute difference in ITN ownership estimates between all households and those with mobile phones. Large differences in ITN ownership estimates between these two groups are especially apparent in earlier DHS surveys when household-level mobile phone ownership was still quite low. However, in later surveys, differences in estimates are much lower.

## Discussion

Our findings show that MPSs may reliably capture ITN coverage estimates when both mobile phone and ITN ownership are high, but they may fail to capture coverage gaps in underserved subpopulations during periods of declining ITN coverage. We report minimal non-coverage bias in ITN coverage estimates between mobile phone-owning households and all households beginning around 2011, when ITN distribution campaigns achieved high coverage and mobile phone ownership was expanding. At this time, mean percentage point differences in ITN coverage indicators between these two groups were low, suggesting that MPS could reliably estimate ITN coverage across the population when both mobile phone ownership and ITN coverage are relatively high. However, as ITN coverage declined between 2011 and 2015, likely due to the absence of mass ITN distribution strategies during this period, mean percentage point differences in ITN coverage estimates began to increase between mobile phone-owning households and non-mobile phone-owning households. This divergence suggests growing non-coverage bias in would-be MPS estimates and may be attributed to higher rates of ITN attrition in households without mobile phones, which are often lower-income and may lack the resources to replace nets as they deteriorate. In contrast, mobile phone-owning households appeared to be better able to replenish or maintain ITNs over time. As mobile phone ownership increased, this subgroup became more representative of the overall sample of households, keeping ITN coverage estimates between mobile phone-owning and all households more closely aligned.

For the three ITN coverage indicators assessed using the 2017 MIS data, national-level estimates did not exceed a 2.5 percentage point difference for mobile phone-owning households compared with the overall sample of households. Differences in regional estimates for these same indicators at the same survey time point rarely exceeded±3 percentage points. In their study assessing the external validity of ITN use estimates collected through a nationally representative random digit dial MPS in Ghana, L’Engle *et al* compared participant responses to the Ghana DHS, finding only a 1.7 percentage point difference.^39^ The authors also found that ITN use estimates among children under 5 years and pregnant women differed by −4.3 and 7.7 percentage points, respectively, between the two survey methodologies.

Results from the current study parallel those of multiple other studies which have reported higher rates of mobile phone ownership among male, younger, higher educated and wealthier respondents in LMIC.^39^,^4149 62 63^ Although MPS may continue to exclude households with specific characteristics, they would presumably do so with decreasing frequency as the percentage of households with mobile phones increases.^47^ At the time of the 2007–2008 AIS-MIS, for example, selection bias for an MPS would have been a much more important consideration than at the time of the 2017 MIS, considering that only 28% of households reported mobile phone ownership in 2007–2008 vs 82% in 2017. However, with the pace of new acquisitions slowing in Tanzania, non-coverage error—and the potential for non-coverage bias—will likely persist among population segments that still lack access to mobile phones. Additionally, mobile phone ownership was a significant predictor of whether a household owned at least one ITN (aOR (95% CI): 1.39 (1.16 to 1.67)). This may indicate that the poorest of households, which are consistently less likely to own a mobile phone, and which would be excluded from an MPS, are also those households lacking ITNs. Indeed, our analysis showed that the top four wealth quintiles all had higher adjusted odds of ITN ownership than the lowest wealth quintile. The precaution being that analysis of MPS data and resultant recommendations, which may be skewed by non-coverage bias, could lead to the differential (or incorrect) implementation of interventions across respondents and non-respondents.^64^

In the context of a real-world study, where inequities in mobile phone ownership persist, the extent to which ownership status continues to serve as a salient differentiator of estimates for ITN coverage indicators remains a question in need of additional research.^48 65^ Namely, if non-mobile phone-owning households are systematically different regarding ITN ownership, MPS results will be biased.^66^ The regression analysis presented in table 2 indicates that mobile phone-owning households were more likely to own an ITN, at least at the time of the 2017 MIS. Furthermore, the observed differences between mobile phone-owning and non-mobile phone-owning households depicted in the lower panels of figure 2 suggest that ITN coverage estimates derived from MPS could be biased, especially in regions or periods without intensive ITN distribution efforts. The two nationwide ITN distribution campaigns (U5CC, UCC1) conducted in Tanzania between 2009 and 2011 appear to have only temporarily reduced differences in ITN coverage between mobile phone-owning and non-mobile phone-owning households, indicating that ITN coverage inequities remain relevant despite relatively widespread mobile phone access.

### Limitations

Mobile phone ownership status was only available at the household level as were the demographic variables used in the regression analyses (eg, head of household sex, head of household age and head of household educational attainment). Thus, the regression model used to predict mobile phone ownership must interpret results through household-level characteristics rather than mobile phone-owner characteristics. The assumption made in this study is that head of household characteristics are ascribed to the owner of the mobile phone within that household, which may be incorrect. Additionally, the survey question concerning mobile phone ownership was modified twice over the course of the study period. Changes in the way a survey question is structured could artificially increase or decrease observed trends, particularly if, prior to or after the 2011–2012 survey, respondents reported ownership even if a mobile phone was non-functional. Our analysis was predicated on household-level ownership of a mobile phone which should mitigate, to a certain degree, issues with measurement error in that we assume individual ownership implies household ownership.

No assumptions are made about the MPS survey modality (ie, interviewer-directed vs self-interviewing methods), each of which carries with it the potential for mode effects, which are described as systematic errors in survey responses attributable to the modality through which the questionnaire is administered. Varying degrees of response bias have been noted between telephone and face-to-face interviews as well as across different MPS modalities and stem from differences in question order, perceived pressure to provide socially desirable responses, propensity for satisficing, as well as acquiescence, primacy and recency effects.^67^,^79^

The analyses presented in this paper assume that either a male or female MPS respondent could provide reliable information on household-level indicators such as bed net ownership and household size. However, evidence from previous studies suggests that male and female co-heads of households often provide different responses to common questions, even on fundamental household details.^80^,^84^ While some of these discrepancies may be due to measurement error, Ambler *et al* highlight a systematic component attributable to asymmetric information within households. This asymmetry could mean that, in some cases, a male respondent may lack precise knowledge of household size or ITN ownership, potentially affecting the accuracy of indicator estimates.

The current study assumed that all households reporting ownership of a mobile phone would have participated in an MPS; however, response rates vary considerably across MPS modality types. Previous random digit dial surveys in LMIC have calculated response rates at 1%,^62^ 19%–40%,^63^ 31%,^39^ 40%^85^ and 61%,^86^ depending on MPS modality, country and offered incentives. While low response rates do not always lead to biased estimates, biased estimates would occur if the propensity for non-response is associated with the measure under consideration.^87^ The effects of non-response bias could not be examined through the use of the DHS data sets. Future work should examine differences in demographic profiles from MPS against population surveys or census results to better understand the impact of non-response.

## Conclusions

Indicators related to ITN coverage provide crucial insight for country malaria programmes, enabling them to assess the overall reach of their distribution activities and plan for future procurement and campaigns based on coverage gaps. Annual monitoring via MPS, for example, could therefore help guide programme strategies more acutely. Note that weighting adjustment schemes (such as poststratification or survey raking) using household-level characteristics (ie, asset ownership) or basic demographic characteristics such as age and sex may help to address coverage bias in MPS due to persistent inequities in mobile phone ownership across LMIC populations, although there is some evidence to suggest that this correction method is not always in the expected direction and is to be used with caution.^3366 88^,^91^

Due to the propensity of MPS to be relatively brief in administration, it is unlikely they will replace traditional face-to-face surveys whose enumerators may spend hours collecting wide-ranging information from a single respondent (as is done for DHS/MIS). However, MPS could serve as an interim or supplemental data collection method capable of providing programme administrators with more frequent monitoring of specific indicators. This notion is particularly relevant given that nationally representative household surveys used to track ITN coverage indicators have historically occurred about once every 2.5 years in Tanzania. Coupled with research demonstrating ITN functional life to be considerably shorter than the oft-assumed 3-year lifespan,^26^,^30^ in addition to recommendations calling for ITN monitoring efforts to begin within 1 year following mass distribution,^92^ carefully designed MPSs seem well positioned to keep country malaria programmes abreast of ITN attrition rates and how they may vary over space and time. As this paper only focused on the potential for non-coverage bias arising from non-mobile phone ownership, additional research is needed particularly for issues related to non-response bias, non-response error and measurement error.

## Supplementary material

10.1136/bmjph-2024-001379 online supplemental figure 1

10.1136/bmjph-2024-001379 online supplemental figure 2

10.1136/bmjph-2024-001379 online supplemental table 1

10.1136/bmjph-2024-001379 online supplemental table 2

10.1136/bmjph-2024-001379 online supplemental table 3

10.1136/bmjph-2024-001379 online supplemental table 4

10.1136/bmjph-2024-001379 online supplemental table 5

## Data Availability

Data are available on reasonable request.

## References

[1] Ministry of Health CD Gender, Elderly and Children-MoHCDGEC/Tanzania Mainland, Ministry of Health - MoH/Zanzibar, National Bureau of Statistics - NBS/Tanzania, Office of Chief Government Statistician - OCGS/Zanzibar, ICF (2016). Tanzania demographic and health survey and malaria indicator survey 2015-2016. http://dhsprogram.com/pubs/pdf/FR321/FR321.pdf.

[2] Ministry of Health, Community Development, Gender E and C (MoHCDGEC) [Tanzania M, Ministry of Health (MoH) [Zanzibar], Tanzania NB of S (NBS), Office of the Chief Government Statistician (OCGS), ICF (2017). Tanzania malaria indicator survey 2017.

[3] Ministry of Health (MoH) [Tanzania Mainland], Ministry of Health (MoH) [Zanzibar], National Bureau of Statistics (NBS), Office of the Chief Government Statistician (OCGS), and ICF (2023). Tanzania demographic and health survey and malaria indicator survey 2022. https://dhsprogram.com/publications/publication-fr382-dhs-final-reports.cfm.

[4] Tanzanian Ministry of Health and Social Welfare. NATNETS - Retired NATNETS - retired. natl. insectic. treat. nets natnets program compon. http://www.natnets.org/.

[5] Hanson K, Nathan R, Marchant T (2008). Vouchers for scaling up insecticide-treated nets in Tanzania: methods for monitoring and evaluation of a national health system intervention. BMC Public Health.

[6] Kramer K, Mandike R, Nathan R (2017). Effectiveness and equity of the Tanzania National Voucher Scheme for mosquito nets over 10 years of implementation. Malar J.

[7] USAID (2019). Tanzania - malaria operational plan fy 2019. https://d1u4sg1s9ptc4z.cloudfront.net/uploads/2021/03/fy-2019-tanzania-malaria-operational-plan.pdf.

[8] Bonner K, Mwita A, McElroy PD (2011). Design, implementation and evaluation of a national campaign to distribute nine million free LLINs to children under five years of age in Tanzania. Malar J.

[9] Renggli S, Mandike R, Kramer K (2013). Design, implementation and evaluation of a national campaign to deliver 18 million free long-lasting insecticidal nets to uncovered sleeping spaces in Tanzania. Malar J.

[10] Lalji S, Ngondi JM, Thawer NG (2016). School Distribution as Keep-Up Strategy to Maintain Universal Coverage of Long-Lasting Insecticidal Nets: Implementation and Results of a Program in Southern Tanzania. Glob Health Sci Pract.

[11] Binka FN, Mensah OA, Mills A (1997). The cost-effectiveness of permethrin impregnated bednets in preventing child mortality in Kassena—Nankana district of Northern Ghana. Health Policy.

[12] Evans DB, Azene G, Kirigia J (1997). Should governments subsidize the use of insecticide-impregnated mosquito nets in Africa? Implications of a cost-effectiveness analysis. Health Policy Plan.

[13] Picard J, Aikins M, Alonso PL (1993). A malaria control trial using insecticide-treated bed nets and targeted chemoprophylaxis in a rural area of The Gambia, West Africa. 8. Cost-effectiveness of bed net impregnation alone or combined with chemoprophylaxis in preventing mortality and morbidity from malaria in Gambian children. Trans R Soc Trop Med Hyg U K.

[14] Aikins MK, Fox-Rushby J, D’Alessandro U (1998). The Gambian National Impregnated Bednet Programme: costs, consequences and net cost-effectiveness. Soc Sci Med.

[15] Goodman CA, Coleman PG, Mills AJ (1999). Cost-effectiveness of malaria control in sub-Saharan Africa. The Lancet.

[16] Vanden Eng JL, Thwing J, Wolkon A (2010). Assessing bed net use and non-use after long-lasting insecticidal net distribution: a simple framework to guide programmatic strategies. Malar J.

[17] Larsen DA, Keating J, Miller J (2010). Barriers to insecticide-treated mosquito net possession 2 years after a mass free distribution campaign in Luangwa District, Zambia. PLoS ONE.

[18] Zhou G, Li JS, Ototo EN (2014). Evaluation of universal coverage of insecticide-treated nets in western Kenya: field surveys. Malar J.

[19] Zegers de Beyl C, Koenker H, Acosta A (2016). Multi-country comparison of delivery strategies for mass campaigns to achieve universal coverage with insecticide-treated nets: what works best?. Malar J.

[20] Aday LA, Cornelius LJ (2011). Designing and Conducting Health Surveys: A Comprehensive Guide.

[21] The Sustainable Development Solutions Network (2015). Data for development: a needs assessment for sdg monitoring and statistical capacity development. https://resources.unsdsn.org/data-for-development-a-needs-assessment-for-sdg-monitoring-and-statistical-capacity-development.

[22] National Bureau of Statistics - NBS/Tanzania, ORC Macro (2005). Tanzania demographic and health survey 2004-2005. http://dhsprogram.com/pubs/pdf/FR173/FR173.pdf.

[23] Tanzania Commission for AIDS - TACAIDS, Zanzibar AIDS Commission - ZAC/Tanzania, National Bureau of Statistics - NBS/Tanzania, Office of the Chief Government Statistician - OCGS/Tanzania, Macro International (2008). Tanzania hiv/aids and malaria indicator survey 2007-08. http://dhsprogram.com/pubs/pdf/AIS6/AIS6.pdf.

[24] National Bureau of Statistics - NBS/Tanzania, ICF Macro Tanzania demographic and health survey 2010. http://dhsprogram.com/pubs/pdf/FR243/FR243.pdf.

[25] (2013). Tanzania hiv/aids and malaria indicator survey 2011-12. Tanzania Commission for AIDS - TACAIDS, Zanzibar AIDS Commission - ZAC/Tanzania, National Bureau of Statistics - NBS/Tanzania, Office of the Chief Government Statistician - OCGS/Tanzania, ICF International.

[26] Gnanguenon V, Azondekon R, Oke-Agbo F (2014). Durability assessment results suggest a serviceable life of two, rather than three, years for the current long-lasting insecticidal (mosquito) net (LLIN) intervention in Benin. BMC Infect Dis.

[27] Mboma ZM, Overgaard HJ, Moore S (2018). Mosquito net coverage in years between mass distributions: a case study of Tanzania, 2013. Malar J.

[28] Massue DJ, Moore SJ, Mageni ZD (2016). Durability of Olyset campaign nets distributed between 2009 and 2011 in eight districts of Tanzania. Malar J.

[29] Bhatt S, Weiss DJ, Mappin B (2015). Coverage and system efficiencies of insecticide-treated nets in Africa from 2000 to 2017. Elife.

[30] Lorenz LM, Bradley J, Yukich J (2020). Comparative functional survival and equivalent annual cost of 3 long-lasting insecticidal net (LLIN) products in Tanzania: A randomised trial with 3-year follow up. PLoS Med.

[31] Hiruy HN, Irish SR, Abdelmenan S (2023). Durability of long-lasting insecticidal nets (LLINs) in Ethiopia. Malar J.

[32] Croke K, Dabalen A, Demombybes G (2012). Collecting High Frequency Panel Data in Africa Using Mobile Phone Interviews. The World Bank.

[33] Mahfoud Z, Ghandour L, Ghandour B (2015). Cell Phone and Face-to-face Interview Responses in Population-based Surveys. Field methods.

[34] Gibson DG, Pereira A, Farrenkopf BA (2017). Mobile Phone Surveys for Collecting Population-Level Estimates in Low- and Middle-Income Countries: A Literature Review. J Med Internet Res.

[35] Hyder AA, Wosu AC, Gibson DG (2017). Noncommunicable Disease Risk Factors and Mobile Phones: A Proposed Research Agenda. J Med Internet Res.

[36] Ballivian A, Azevedo JP, Durbin W (2015). Using mobile phones for high-frequency data collection.

[37] Vecino-Ortiz AI, Nagarajan M, Katumba KR (2021). A cost study for mobile phone health surveys using interactive voice response for assessing risk factors of noncommunicable diseases. Popul Health Metr.

[38] Al Kibria GM, Kagoro F, Pariyo G (2024). A nationwide mobile phone survey for tobacco use in Tanzania: Sample quality and representativeness compared to a household survey. Prev Med Rep.

[39] L’Engle K, Sefa E, Adimazoya EA (2018). Survey research with a random digit dial national mobile phone sample in Ghana: Methods and sample quality. PLoS ONE.

[40] Pew Research Center (2018). Internet connectivity seen as having positive impact on life in sub-saharan africa. https://www.pewresearch.org/global/2018/10/09/internet-connectivity-seen-as-having-positive-impact-on-life-in-sub-saharan-africa/.

[41] GSMA (2018). The mobile gender gap report 2018. https://www.gsma.com/mobilefordevelopment/connected-women/the-mobile-gender-gap-report-2018/.

[42] Biscaye P, Goddard J, Lane M (2015). Review of mobile coverage.

[43] GSMA (2017). The mobile economy sub-saharan africa 2017. https://www.gsma.com/subsaharanafrica/resources/the-mobile-economy-2017.

[44] Mtenzi F, Chachage B, Ngumbuke F (2018). The Growth of Tanzanian Mobile Phone Sector: Triumph of Quantity, Failure of Quality?.

[45] GSMA (2022). The mobile economy sub-saharan africa 2022. https://www.gsma.com/mobileeconomy/sub-saharan-africa/.

[46] Elkasabi M, Khan A (2023). The Evolution of Mobile Phone Surveys in Low- and Middle-Income Countries: A Study of Coverage Structure. Int J Public Opin Res.

[47] Leo B, Morello R, Mellon J (2015). Do Mobile Phone Surveys Work in Poor Countries?. SSRN Journal.

[48] Tran MC, Labrique AB, Mehra S (2015). Analyzing the mobile “digital divide”: changing determinants of household phone ownership over time in rural bangladesh. JMIR Mhealth Uhealth.

[49] Rheault M, McCarthy J (2016). Disparities in cellphone ownership pose challenges in africa. https://news.gallup.com/poll/189269/disparities-cellphone-ownership-pose-challenges-africa.aspx.

[50] GSMA (2018). The mobile economy sub-saharan africa 2018. https://data.gsmaintelligence.com/research/research/research-2018/the-mobile-economy-sub-saharan-africa-2018.

[51] Lau CQ, Lombaard A, Baker M (2019). How Representative Are SMS Surveys in Africa? Experimental Evidence From Four Countries. Int J Public Opin Res.

[52] R Core Team (2018). R: a language and environment for statistical computing.

[53] Staveteig Ford S, Mallick L (2014). Intertemporal Comparisons of Poverty and Wealth with DHS Data: A Harmonized Asset Index Approach.

[54] Rue H, Lindgren F, Simpson D (2019). INLA: Full Bayesian Analysis of Latent Gaussian Models Using Integrated Nested Laplace Approximations.

[55] Toh KB, Bliznyuk N, Valle D (2021). Improving national level spatial mapping of malaria through alternative spatial and spatio-temporal models. Spat Spatiotemporal Epidemiol.

[56] Pesaresi M, Politis P (2023). GHS-BUILT-S R2023A - GHS Built-up Surface Grid, Derived from Sentinel2 Composite and Landsat, Multitemporal (1975-2030).

[57] Lloyd CT, Chamberlain H, Kerr D (2019). Global spatio-temporally harmonised datasets for producing high-resolution gridded population distribution datasets. Big Earth Data.

[58] Friedl M, Sulla-Menashe D (2022). MODIS/terra+aqua land cover type yearly l3 global 500m sin grid v061.

[59] Institute for Health Metrics and Evaluation (IHME) Africa educational attainment geospatial estimates 2000-2015.

[60] Graetz N, Friedman J, Osgood-Zimmerman A (2018). Mapping local variation in educational attainment across Africa. Nature New Biol.

[61] Tusting LS, Bisanzio D, Alabaster G (2019). Mapping changes in housing in sub-Saharan Africa from 2000 to 2015. Nature New Biol.

[62] Pariyo GW, Greenleaf AR, Gibson DG (2019). Does mobile phone survey method matter? Reliability of computer-assisted telephone interviews and interactive voice response non-communicable diseases risk factor surveys in low and middle income countries. PLoS ONE.

[63] Gibson DG, Wosu AC, Pariyo GW (2019). Effect of airtime incentives on response and cooperation rates in non-communicable disease interactive voice response surveys: randomised controlled trials in Bangladesh and Uganda. BMJ Glob Health.

[64] Ali J, Labrique AB, Gionfriddo K (2017). Ethics Considerations in Global Mobile Phone-Based Surveys of Noncommunicable Diseases: A Conceptual Exploration. J Med Internet Res.

[65] Carter A, Liddle J, Hall W (2015). Mobile Phones in Research and Treatment: Ethical Guidelines and Future Directions. JMIR Mhealth Uhealth.

[66] Lee S, Brick JM, Brown ER (2010). Growing cell-phone population and noncoverage bias in traditional random digit dial telephone health surveys. Health Serv Res.

[67] Jordan LA, Marcus AC, Reeder LG (1980). Response Styles in Telephone and Household Interviewing: A Field Experiment. Public Opin Q.

[68] Dillman DA, Robert GM (1984). The influence of survey method on question response.

[69] McCLENDON MJ (1991). Acquiescence and Recency Response-Order Effects in Interview Surveys. Sociol Methods Res.

[70] Schwarz N, Strack F, Hippler H-J (1991). The impact of administration mode on response effects in survey measurement. Appl Cogn Psychol.

[71] Leeuw D, Desiree E (1992). Data quality in mail, telephone and face to face surveys. https://eric.ed.gov/?id=ED374136.

[72] Tourangeau R, Rips LJ, Rasinski K (2000). The Psychology of Survey Response.

[73] Holbrook AL, Green MC, Krosnick JA (2003). Telephone versus Face-to-Face Interviewing of National Probability Samples with Long Questionnaires: Comparisons of Respondent Satisficing and Social Desirability Response Bias. Public Opin Q.

[74] Lynn P, Jackle A, Roberts C (2006). Telephone versus face-to-face interviewing: mode effects on data quality and likely causes: report on phase II of the ESS-Gallup mixed mode methodology project. ISER Work Pap Ser.

[75] Lavrakas PJ, Shuttles CD, Steeh C (2007). The State of Surveying Cell Phone Numbers in the United States: 2007 and Beyond. Public Opin Q.

[76] Ongena YP, Dijkstra W (2007). A model of cognitive processes and conversational principles in survey interview interaction. Appl Cogn Psychol.

[77] Kreuter F, Presser S, Tourangeau R (2008). Social Desirability Bias in CATI, IVR, and Web Surveys: The Effects of Mode and Question Sensitivity. Public Opin Q.

[78] Hyman IE, Boss SM, Wise BM (2010). Did you see the unicycling clown? Inattentional blindness while walking and talking on a cell phone. Appl Cogn Psychol.

[79] Vogl S (2013). Telephone Versus Face-to-Face Interviews: Mode Effect on Semistructured Interviews with Children. Sociol Methodol.

[80] Acosta M, van Wessel M, van Bommel S (2020). What does it Mean to Make a ‘Joint’ Decision? Unpacking Intra-household Decision Making in Agriculture: Implications for Policy and Practice. J Dev Stud.

[81] Ambler K, Doss C, Kieran C (2021). He Says, She Says: Spousal Disagreement in Survey Measures of Bargaining Power. Econ Dev Cult Change.

[82] Anderson CL, Reynolds TW, Gugerty MK (2017). Husband and Wife Perspectives on Farm Household Decision-making Authority and Evidence on Intra-household Accord in Rural Tanzania. World Dev.

[83] Annan J, Donald A, Goldstein M (2021). Taking power: Women’s empowerment and household Well-being in Sub-Saharan Africa. World Dev.

[84] Twyman J, Useche P, Deere CD (2015). Gendered Perceptions of Land Ownership and Agricultural Decision-making in Ecuador: Who Are the Farm Managers?. Land Econ.

[85] Larmarange J, Kassoum O, Kakou É (2016). Faisabilité et représentativité d’une enquête téléphonique avec échantillonnage aléatoire de lignes mobiles en Côte d’Ivoire. Population (Paris).

[86] Islam K, Rahman M, Sharif A (2013). Introducing mobile phone for interview in surveillance system in bangladesh: validation of the method. https://www.astmh.org/ASTMH/media/Documents/AbstractBook2013Final.pdf.

[87] AAPOR Cell Phone Task Force (2010). New Considerations for Survey Researchers When Planning and Conducting RDD Telephone Surveys in the U.S. With Respondents Reached via Cell Phone Numbers.

[88] Lau CQ, Cronberg A, Marks L (2019). In Search of the Optimal Mode for Mobile Phone Surveys in Developing Countries. A Comparison of IVR, SMS, and CATI in Nigeria. Surv Res Methods.

[89] Greenleaf AR, Gadiaga A, Guiella G (2020). Comparability of modern contraceptive use estimates between a face-to-face survey and a cellphone survey among women in Burkina Faso. PLoS ONE.

[90] Ahmed S, Romero-Prieto J, Sánchez-Páez DA (2024). Sample selection bias in adult mortality estimates from mobile phone surveys: Evidence from 25 low- and middle-income countries. DemRes.

[91] Brubaker J, Kilic T, Wollburg P (2021). Representativeness of individual-level data in COVID-19 phone surveys: Findings from Sub-Saharan Africa. PLoS ONE.

[92] Wills AB, Smith SC, Anshebo GY (2013). Physical durability of PermaNet 2.0 long-lasting insecticidal nets over three to 32 months of use in Ethiopia. Malar J.

