# Supplementary material for: Assessing trends in non-coverage bias in mobile phone surveys for estimating insecticide-treated net coverage: a cross-sectional analysis in Tanzania, 2007–2017
Source: BMJ Public Health. 2025 Mar 4;3(1):e001379. doi: 10.1136/bmjph-2024-001379 (PMC11883883; doi:10.1136/bmjph-2024-001379)
Supplement: online supplemental figure 1 [file bmjph-3-1-s001.pdf]

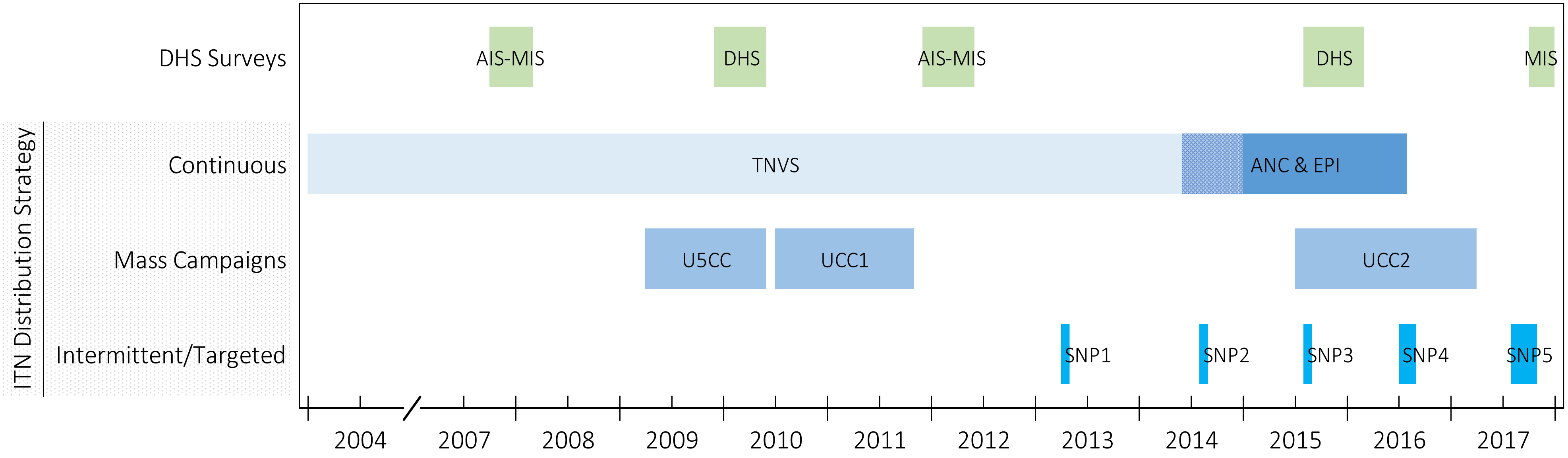

Supplemental Figure 1. Timeline of DHS surveys and ITN distribution strategies up to the 2017 MIS. Note that the SNP was implemented as part of a 'keep up' ITN distribution strategy and was active on a non-continuous basis.

ITN Insecticide Treated Net, AIS HIV/AIDS Indicator Survey, DHS Demographic and Health Survey, MIS Malaria Indicator Survey, TNVS Tanzania National ITN Voucher Scheme, ANC Antenatal Care, EPI Expanded Program on Immunizations, U5CC Under-5 Coverage Campaign, U5CC Under-5 Coverage Campaign, UCC1 Universal Coverage Campaign One, UCC2 Universal Coverage Campaign Two, SNP School Net Program
