# Supplementary material for: Assessing trends in non-coverage bias in mobile phone surveys for estimating insecticide-treated net coverage: a cross-sectional analysis in Tanzania, 2007–2017
Source: BMJ Public Health. 2025 Mar 4;3(1):e001379. doi: 10.1136/bmjph-2024-001379 (PMC11883883; doi:10.1136/bmjph-2024-001379)
Supplement: online supplemental figure 2 [file bmjph-3-1-s002.pdf]

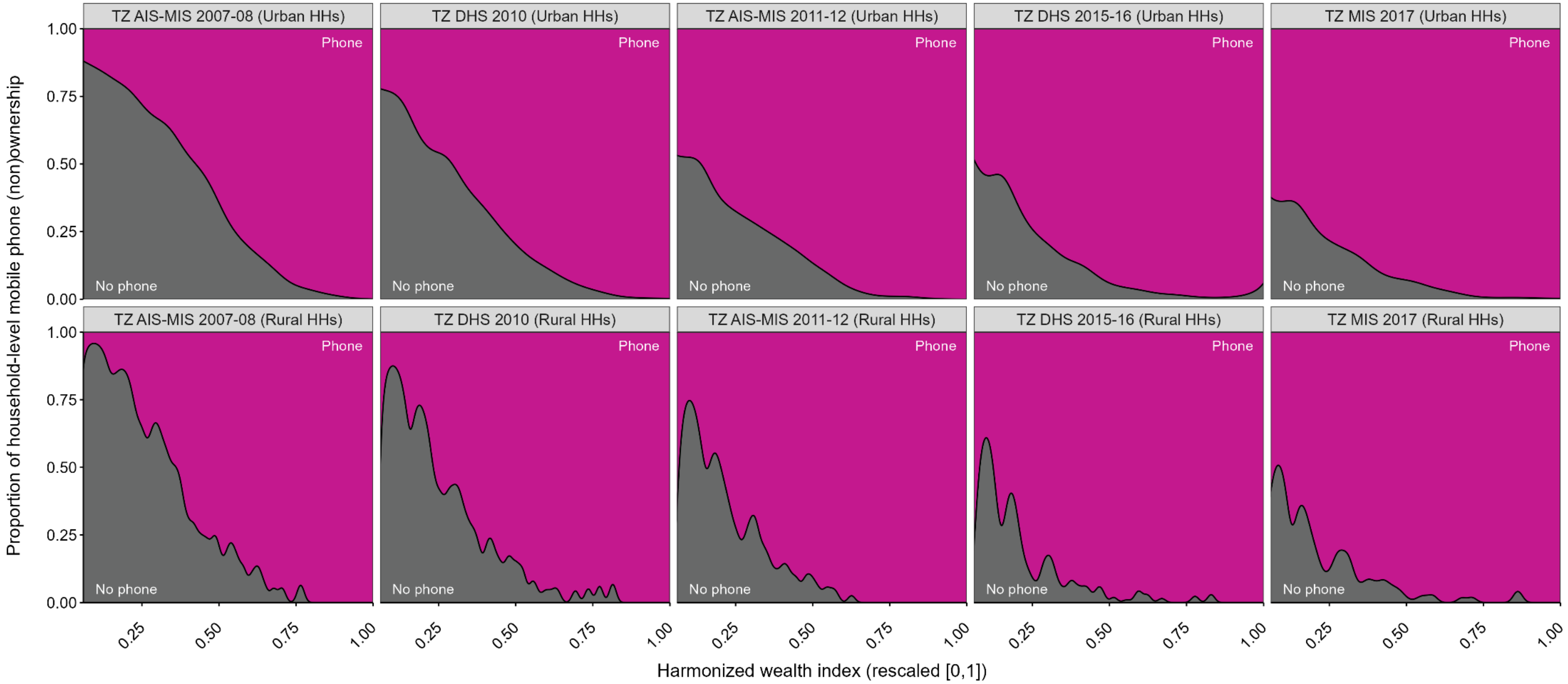

Supplemental Figure 2. Conditional density plots of mobile phone ownership status by harmonized wealth index, location of residence, and survey year.

TZ Tanzania, *AIS HIV/AIDS* Indicator Survey, *DHS* Demographic and Health Survey, *MIS* Malaria Indicator Survey, *HHs* households
