# Supplementary material for: Assessing trends in non-coverage bias in mobile phone surveys for estimating insecticide-treated net coverage: a cross-sectional analysis in Tanzania, 2007–2017
Source: BMJ Public Health. 2025 Mar 4;3(1):e001379. doi: 10.1136/bmjph-2024-001379 (PMC11883883; doi:10.1136/bmjph-2024-001379)
Supplement: online supplemental table 1 [file bmjph-3-1-s003.pdf]

Supplemental Table 1. TZ AIS-MIS 2007-08. Households or household population by RBM-MERG ITN indicator, region, and mobile phone ownership status. Point estimates from bootstrapping method of resampling.

	Households with at least one ITN			Households with at least one ITN for every two people			Population with access to an ITN in their household		
Region	Households with mobile phones % (N)	Households without mobile phones % (N)	All households % (N)	Households with mobile phones % (N)	Households without mobile phones % (N)	All households % (N)	Population in households with mobile phones % (N)	Population in households without mobile phones % (N)	Population among households % (N)
**National**	66.7 (2626)	37.8 (5868)	46.8 (8494)	30.2 (2626)	13.1 (5868)	18.4 (8494)	48.9 (14344)	25.5 (29140)	33.2 (43484)
Arusha	52.6 (133)	18.1 (160)	33.8 (293)	28.6 (133)	4.4 (160)	15.4 (293)	38.7 (586)	9.3 (738)	22.4 (1324)
Dar es Salaam	72.3 (278)	61.7 (86)	69.8 (364)	36.7 (278)	33.7 (86)	36.0 (364)	56.3 (1260)	42.4 (292)	53.7 (1552)
Dodoma	60.6 (61)	23.2 (232)	31.0 (293)	21.2 (61)	5.6 (232)	8.9 (293)	41.4 (297)	15.1 (1007)	21.1 (1304)
Katavi/Rukwa	59.2 (49)	26.9 (253)	32.1 (302)	22.6 (49)	5.5 (253)	8.3 (302)	44.0 (259)	14.8 (1375)	19.4 (1634)
Kigoma	44.8 (47)	28.8 (264)	31.2 (311)	17.1 (47)	3.8 (264)	5.8 (311)	26.5 (257)	15.3 (1469)	16.9 (1726)
Kilimanjaro	36.9 (141)	16.8 (172)	25.8 (313)	10.6 (141)	5.2 (172)	7.7 (313)	21.4 (738)	9.6 (783)	15.3 (1521)
Lake zone*	60.5 (213)	31.9 (701)	38.6 (914)	25.8 (213)	6.4 (701)	10.9 (914)	36.9 (1368)	16.9 (3993)	22.0 (5361)
Lindi	64.3 (42)	37.6 (274)	41.1 (316)	35.8 (42)	18.6 (274)	20.9 (316)	49.2 (183)	30.4 (1085)	33.1 (1268)
Manyara	36.5 (52)	16.1 (248)	19.6 (300)	11.5 (52)	2.0 (248)	3.7 (300)	21.5 (261)	7.7 (1371)	9.9 (1632)
Mara	70.2 (94)	46.2 (210)	53.6 (304)	20.2 (94)	9.6 (210)	12.8 (304)	40.1 (593)	22.6 (1298)	28.1 (1891)
Morogoro	73.6 (83)	33.0 (200)	44.8 (283)	44.6 (83)	12.5 (200)	21.9 (283)	55.9 (361)	21.7 (807)	32.3 (1168)
Mtwara	62.1 (29)	40.3 (272)	42.5 (301)	31.0 (29)	19.1 (272)	20.2 (301)	46.6 (118)	29.9 (1067)	31.6 (1185)
Njombe/Iringa	39.4 (61)	12.9 (248)	18.1 (309)	6.6 (61)	4.0 (248)	4.5 (309)	20.9 (316)	10.1 (1013)	12.6 (1329)
Pemba North	83.1 (231)	67.5 (397)	73.2 (628)	41.6 (231)	28.5 (397)	33.3 (628)	66.6 (1404)	55.6 (2106)	60.0 (3510)
Pemba South	87.6 (250)	74.3 (381)	79.6 (631)	39.7 (250)	32.0 (381)	35.0 (631)	67.0 (1565)	57.5 (2106)	61.6 (3671)
Pwani	66.2 (68)	40.5 (183)	47.4 (251)	29.3 (68)	15.8 (183)	19.5 (251)	49.0 (330)	29.0 (761)	35.1 (1091)
Ruvuma	68.3 (41)	35.2 (259)	39.7 (300)	39.1 (41)	8.5 (259)	12.7 (300)	44.6 (211)	20.7 (1316)	24.0 (1527)
Singida	56.8 (37)	23.6 (254)	27.8 (291)	18.9 (37)	3.5 (254)	5.5 (291)	37.0 (192)	11.1 (1235)	14.6 (1427)
Songwa/Mbeya	46.7 (77)	24.4 (230)	30.0 (307)	14.3 (77)	9.6 (230)	10.8 (307)	34.8 (400)	17.4 (964)	22.4 (1364)
Tabora	70.9 (55)	32.0 (219)	39.8 (274)	32.6 (55)	6.8 (219)	12.0 (274)	42.2 (332)	15.2 (1515)	20.0 (1847)
Tanga	61.6 (86)	31.0 (219)	39.6 (305)	25.7 (86)	11.4 (219)	15.4 (305)	45.8 (406)	19.1 (984)	26.9 (1390)
Zanzibar North	80.8 (104)	65.5 (200)	70.7 (304)	40.3 (104)	26.0 (200)	30.9 (304)	64.5 (584)	50.2 (942)	55.7 (1526)
Zanzibar Sth/Cntrl	75.4 (163)	68.7 (144)	72.3 (307)	36.8 (163)	36.1 (144)	36.5 (307)	55.6 (951)	59.1 (616)	57.0 (1567)
Zanzibar West	68.4 (231)	61.3 (62)	66.9 (293)	29.8 (231)	29.1 (62)	29.7 (293)	52.2 (1372)	45.8 (297)	51.0 (1669)

N indicates the total number of households or household population in each category.

*Geita, Shinyanga, Mwanza, Kagera, and Simiyu were grouped into a single entity (Lake Zone).
