# Supplementary material for: Assessing trends in non-coverage bias in mobile phone surveys for estimating insecticide-treated net coverage: a cross-sectional analysis in Tanzania, 2007–2017
Source: BMJ Public Health. 2025 Mar 4;3(1):e001379. doi: 10.1136/bmjph-2024-001379 (PMC11883883; doi:10.1136/bmjph-2024-001379)
Supplement: online supplemental table 2 [file bmjph-3-1-s004.pdf]

Supplemental Table 2. TZ DHS 2010. Households or household population by RBM-MERG ITN indicator, region, and mobile phone ownership status. Point estimates from bootstrapping method of resampling.

	Households with at least one ITN			Households with at least one ITN for every two people			Population with access to an ITN in their household		
Region	Households with mobile phones % (N)	Households without mobile phones % (N)	All households % (N)	Households with mobile phones % (N)	Households without mobile phones % (N)	All households % (N)	Population in households with mobile phones % (N)	Population in households without mobile phones % (N)	Population among households % (N)
**National**	72.2 (4546)	60.2 (5072)	65.9 (9618)	29.1 (4546)	19.1 (5072)	23.8 (9618)	53.1 (25058)	43.6 (23808)	48.5 (48866)
Arusha	52.5 (198)	37.5 (160)	45.8 (358)	19.2 (198)	6.2 (160)	13.4 (358)	38.1 (921)	22.0 (738)	30.9 (1659)
Dar es Salaam	61.3 (338)	54.6 (44)	60.4 (382)	30.5 (338)	25.2 (44)	29.8 (382)	45.6 (1377)	36.5 (126)	44.9 (1503)
Dodoma	86.6 (82)	67.9 (296)	72.0 (378)	31.7 (82)	15.8 (296)	19.3 (378)	59.6 (419)	45.9 (1296)	49.3 (1715)
Katavi/Rukwa	72.1 (107)	64.7 (258)	66.9 (365)	23.4 (107)	14.7 (258)	17.3 (365)	47.7 (551)	43.3 (1308)	44.6 (1859)
Kigoma	66.0 (100)	53.9 (249)	57.3 (349)	18.1 (100)	9.7 (249)	12.1 (349)	46.0 (592)	36.1 (1245)	39.2 (1837)
Kilimanjaro	51.0 (249)	37.7 (127)	46.5 (376)	16.1 (249)	11.0 (127)	14.3 (376)	35.5 (1065)	28.8 (427)	33.6 (1492)
Lake zone*	81.4 (479)	72.4 (640)	76.2 (1119)	27.1 (479)	17.5 (640)	21.6 (1119)	56.1 (3267)	49.7 (3510)	52.8 (6777)
Lindi	74.0 (96)	59.4 (256)	63.4 (352)	33.3 (96)	25.0 (256)	27.3 (352)	56.0 (414)	49.5 (1003)	51.5 (1417)
Manyara	75.0 (132)	70.3 (216)	72.1 (348)	28.0 (132)	17.1 (216)	21.3 (348)	54.0 (704)	49.9 (1091)	51.5 (1795)
Mara	89.8 (167)	79.3 (198)	84.1 (365)	26.9 (167)	17.7 (198)	21.9 (365)	63.4 (1137)	52.4 (1064)	58.1 (2201)
Morogoro	53.3 (169)	23.7 (199)	37.3 (368)	19.5 (169)	10.0 (199)	14.4 (368)	37.2 (845)	13.7 (856)	25.3 (1701)
Mtwara	85.4 (89)	56.9 (269)	64.0 (358)	46.1 (89)	20.1 (269)	26.5 (358)	66.1 (430)	43.9 (1086)	50.2 (1516)
Njombe/Iringa	58.4 (161)	47.1 (206)	52.1 (367)	18.7 (161)	8.2 (206)	12.8 (367)	41.8 (723)	34.6 (817)	38.0 (1540)
Pemba North	86.1 (208)	89.3 (168)	87.5 (376)	36.6 (208)	50.0 (168)	42.6 (376)	67.5 (1336)	73.4 (861)	69.8 (2197)
Pemba South	74.8 (210)	70.0 (160)	72.7 (370)	23.8 (210)	32.5 (160)	27.6 (370)	50.2 (1424)	53.4 (852)	51.3 (2276)
Pwani	80.2 (177)	54.7 (168)	67.8 (345)	30.0 (177)	19.6 (168)	24.9 (345)	58.1 (943)	42.4 (757)	51.1 (1700)
Ruvuma	84.8 (112)	63.2 (266)	69.6 (378)	36.7 (112)	15.4 (266)	21.7 (378)	64.0 (539)	41.5 (1193)	48.5 (1732)
Singida	49.2 (126)	29.8 (255)	36.2 (381)	17.4 (126)	7.0 (255)	10.5 (381)	30.2 (784)	19.7 (1248)	23.8 (2032)
Songwa/Mbeya	62.7 (166)	52.1 (211)	56.8 (377)	22.3 (166)	14.2 (211)	17.8 (377)	45.7 (919)	38.2 (888)	42.0 (1807)
Tabora	84.8 (164)	62.5 (216)	72.1 (380)	32.3 (164)	15.7 (216)	22.9 (380)	54.4 (1093)	40.1 (1333)	46.5 (2426)
Tanga	73.8 (183)	47.3 (186)	60.4 (369)	37.7 (183)	15.0 (186)	26.3 (369)	55.8 (850)	32.2 (692)	45.2 (1542)
Zanzibar North	88.1 (211)	86.5 (178)	87.4 (389)	51.6 (211)	53.4 (178)	52.4 (389)	73.7 (1181)	70.7 (847)	72.4 (2028)
Zanzibar Sth/Cntrl	91.1 (269)	86.6 (112)	89.8 (381)	49.1 (269)	57.1 (112)	51.5 (381)	74.4 (1438)	73.2 (433)	74.1 (1871)
Zanzibar West	61.8 (353)	55.8 (34)	61.3 (387)	22.9 (353)	23.3 (34)	23.0 (387)	43.4 (2106)	48.2 (137)	43.7 (2243)

N indicates the total number of households or household population in each category.

*Geita, Shinyanga, Mwanza, Kagera, and Simiyu were grouped into a single entity (Lake Zone).
