# Supplementary material for: Assessing trends in non-coverage bias in mobile phone surveys for estimating insecticide-treated net coverage: a cross-sectional analysis in Tanzania, 2007–2017
Source: BMJ Public Health. 2025 Mar 4;3(1):e001379. doi: 10.1136/bmjph-2024-001379 (PMC11883883; doi:10.1136/bmjph-2024-001379)
Supplement: online supplemental table 3 [file bmjph-3-1-s005.pdf]

Supplemental Table 3. TZ AIS-MIS 2011-12. Households or household population by RBM-MERG ITN indicator, region, and mobile phone ownership status. Point estimates from bootstrapping method of resampling.

	Households with at least one ITN			Households with at least one ITN for every two people			Population with access to an ITN in their household		
Region	Households with mobile phones % (N)	Households without mobile phones % (N)	All households % (N)	Households with mobile phones % (N)	Households without mobile phones % (N)	All households % (N)	Population in households with mobile phones % (N)	Population in households without mobile phones % (N)	Population among households % (N)
**National**	89.9 (6223)	89.1 (3800)	89.6 (10023)	51.6 (6223)	52.2 (3800)	51.9 (10023)	72.9 (34418)	71.6 (17770)	72.4 (52188)
Arusha	83.7 (269)	77.4 (71)	82.4 (340)	49.5 (269)	32.4 (71)	45.9 (340)	69.4 (1166)	57.6 (290)	67.0 (1456)
Dar es Salaam	78.0 (479)	75.6 (41)	77.9 (520)	54.3 (479)	58.4 (41)	54.6 (520)	71.6 (1904)	72.1 (104)	71.6 (2008)
Dodoma	94.1 (135)	90.9 (210)	92.2 (345)	60.0 (135)	57.6 (210)	58.5 (345)	80.5 (719)	75.5 (905)	77.7 (1624)
Katavi/Rukwa	87.2 (289)	86.3 (337)	86.7 (626)	41.5 (289)	36.5 (337)	38.8 (626)	64.4 (1750)	61.8 (1677)	63.1 (3427)
Kigoma	98.2 (169)	90.6 (180)	94.3 (349)	47.9 (169)	39.9 (180)	43.8 (349)	80.4 (1106)	67.9 (1006)	74.4 (2112)
Kilimanjaro	96.2 (239)	89.7 (107)	94.2 (346)	63.6 (239)	63.6 (107)	63.6 (346)	81.3 (1095)	77.9 (430)	80.3 (1525)
Lake zone*	94.1 (973)	92.3 (730)	93.4 (1703)	39.9 (973)	44.4 (730)	41.8 (1703)	68.6 (6735)	68.5 (4014)	68.5 (10749)
Lindi	96.6 (174)	95.5 (176)	96.0 (350)	79.9 (174)	81.8 (176)	80.8 (350)	90.4 (711)	90.5 (629)	90.5 (1340)
Manyara	93.0 (214)	78.7 (131)	87.5 (345)	42.0 (214)	32.9 (131)	38.5 (345)	67.6 (1282)	61.5 (688)	65.5 (1970)
Mara	96.7 (214)	93.7 (127)	95.6 (341)	42.5 (214)	49.6 (127)	45.2 (341)	73.0 (1516)	77.3 (664)	74.3 (2180)
Morogoro	92.2 (230)	89.7 (107)	91.4 (337)	60.8 (230)	67.3 (107)	62.9 (337)	79.0 (1140)	80.6 (422)	79.4 (1562)
Mtwara	95.1 (163)	89.9 (179)	92.4 (342)	81.6 (163)	73.7 (179)	77.5 (342)	92.0 (707)	81.3 (646)	86.8 (1353)
Njombe/Iringa	94.3 (424)	89.4 (265)	92.5 (689)	63.0 (424)	64.2 (265)	63.4 (689)	83.2 (2007)	79.1 (1011)	81.8 (3018)
Pemba North	85.0 (208)	79.6 (49)	84.0 (257)	51.9 (208)	59.1 (49)	53.3 (257)	71.3 (1226)	68.8 (234)	70.9 (1460)
Pemba South	86.4 (214)	66.7 (48)	82.8 (262)	31.3 (214)	31.2 (48)	31.3 (262)	62.3 (1239)	47.4 (226)	60.0 (1465)
Pwani	95.8 (212)	95.0 (100)	95.5 (312)	68.9 (212)	68.0 (100)	68.6 (312)	84.6 (1063)	83.1 (434)	84.2 (1497)
Ruvuma	94.3 (174)	94.3 (177)	94.3 (351)	62.6 (174)	70.0 (177)	66.4 (351)	82.1 (949)	84.8 (843)	83.4 (1792)
Singida	93.6 (157)	95.4 (197)	94.6 (354)	59.8 (157)	53.3 (197)	56.2 (354)	75.3 (948)	76.7 (1004)	76.0 (1952)
Songwa/Mbeya	94.6 (221)	85.9 (128)	91.4 (349)	68.8 (221)	52.4 (128)	62.8 (349)	82.5 (1186)	71.6 (539)	79.1 (1725)
Tabora	95.0 (221)	92.3 (155)	93.9 (376)	45.2 (221)	53.6 (155)	48.7 (376)	75.1 (1463)	73.5 (728)	74.5 (2191)
Tanga	93.5 (184)	88.1 (151)	91.1 (335)	54.9 (184)	37.7 (151)	47.1 (335)	75.8 (950)	62.7 (748)	70.0 (1698)
Zanzibar North	79.4 (184)	71.0 (83)	76.8 (267)	41.3 (184)	43.3 (83)	41.9 (267)	63.7 (1064)	60.1 (346)	62.8 (1410)
Zanzibar Sth/Cntrl	79.0 (228)	72.8 (37)	78.1 (265)	40.8 (228)	48.4 (37)	41.9 (265)	63.2 (1124)	64.4 (132)	63.3 (1256)
Zanzibar West	69.4 (248)	57.2 (14)	68.7 (262)	37.1 (248)	28.4 (14)	36.6 (262)	54.1 (1368)	45.9 (50)	53.8 (1418)

N indicates the total number of households or household population in each category.

*Geita, Shinyanga, Mwanza, Kagera, and Simiyu were grouped into a single entity (Lake Zone).
