# Supplementary material for: Assessing trends in non-coverage bias in mobile phone surveys for estimating insecticide-treated net coverage: a cross-sectional analysis in Tanzania, 2007–2017
Source: BMJ Public Health. 2025 Mar 4;3(1):e001379. doi: 10.1136/bmjph-2024-001379 (PMC11883883; doi:10.1136/bmjph-2024-001379)
Supplement: online supplemental table 4 [file bmjph-3-1-s006.pdf]

**Supplemental Table 4.** TZ DHS 2015-16. Households or household population by RBM-MERG ITN indicator, region, and mobile phone ownership status. Point estimates from bootstrapping method of resampling.

| | Households with at least one ITN | | | Households with at least one ITN for every two people | | | Population with access to an ITN in their household | | |
|---|---|---|---|---|---|---|---|---|---|
| Region | Households with mobile phones % (N) | Households without mobile phones % (N) | All households % (N) | Households with mobile phones % (N) | Households without mobile phones % (N) | All households % (N) | Population in households with mobile phones % (N) | Population in households without mobile phones % (N) | Population among households % (N) |
| **National** | 70.3 (9950) | 55.6 (2613) | 67.2 (12563) | 38.9 (9950) | 35.3 (2613) | 38.2 (12563) | 58.3 (51886) | 48.6 (10629) | 56.6 (62515) |
| Arusha | 48.3 (356) | 16.0 (62) | 43.6 (418) | 21.1 (356) | 6.4 (62) | 18.9 (418) | 35.1 (1565) | 9.7 (258) | 31.6 (1823) |
| Dar es Salaam | 66.8 (678) | 77.2 (22) | 67.1 (700) | 33.9 (678) | 36.5 (22) | 34.0 (700) | 53.1 (2764) | 61.3 (80) | 53.3 (2844) |
| Dodoma | 48.9 (258) | 21.6 (162) | 38.3 (420) | 20.2 (258) | 4.9 (162) | 14.3 (420) | 31.0 (1206) | 14.2 (608) | 25.4 (1814) |
| Katavi/Rukwa | 71.6 (535) | 39.5 (302) | 60.0 (837) | 36.8 (535) | 24.5 (302) | 32.4 (837) | 57.8 (3052) | 29.5 (1301) | 49.3 (4353) |
| Kigoma | 93.1 (307) | 94.4 (125) | 93.5 (432) | 70.3 (307) | 72.9 (125) | 71.1 (432) | 86.3 (1748) | 83.7 (614) | 85.6 (2362) |
| Kilimanjaro | 66.6 (398) | 51.0 (51) | 64.8 (449) | 39.9 (398) | 33.3 (51) | 39.2 (449) | 53.9 (1576) | 38.0 (158) | 52.5 (1734) |
| Lake zone* | 90.0 (1615) | 91.6 (502) | 90.4 (2117) | 54.5 (1615) | 62.3 (502) | 56.4 (2117) | 77.2 (10488) | 80.1 (2432) | 77.8 (12920) |
| Lindi | 76.0 (317) | 51.8 (106) | 70.0 (423) | 43.9 (317) | 31.1 (106) | 40.7 (423) | 62.5 (1359) | 42.7 (363) | 58.4 (1722) |
| Manyara | 28.8 (323) | 13.6 (103) | 25.1 (426) | 10.2 (323) | 6.8 (103) | 9.4 (426) | 17.5 (1716) | 9.4 (468) | 15.8 (2184) |
| Mara | 93.3 (346) | 85.9 (92) | 91.8 (438) | 59.8 (346) | 58.7 (92) | 59.6 (438) | 80.9 (2100) | 78.3 (475) | 80.4 (2575) |
| Morogoro | 63.9 (285) | 33.6 (113) | 55.3 (398) | 31.2 (285) | 17.6 (113) | 27.4 (398) | 48.6 (1349) | 22.7 (370) | 43.0 (1719) |
| Mtwara | 67.7 (288) | 45.9 (122) | 61.2 (410) | 40.6 (288) | 31.9 (122) | 38.0 (410) | 56.6 (1206) | 45.4 (377) | 53.9 (1583) |
| Njombe/Iringa | 49.4 (634) | 33.7 (187) | 45.8 (821) | 28.9 (634) | 21.4 (187) | 27.2 (821) | 39.3 (2693) | 26.4 (614) | 36.9 (3307) |
| Pemba North | 81.2 (282) | 67.5 (40) | 79.5 (322) | 42.9 (282) | 42.5 (40) | 42.9 (322) | 63.4 (1597) | 51.1 (174) | 62.2 (1771) |
| Pemba South | 78.7 (286) | 79.9 (40) | 78.8 (326) | 38.9 (286) | 37.4 (40) | 38.6 (326) | 58.7 (1657) | 54.2 (179) | 58.3 (1836) |
| Pwani | 66.2 (337) | 43.6 (48) | 63.4 (385) | 35.0 (337) | 27.0 (48) | 34.0 (385) | 51.0 (1464) | 35.4 (147) | 49.6 (1611) |
| Ruvuma | 72.1 (323) | 51.2 (111) | 66.8 (434) | 41.2 (323) | 27.8 (111) | 37.8 (434) | 59.2 (1431) | 42.0 (398) | 55.4 (1829) |
| Singida | 45.8 (332) | 30.2 (106) | 42.0 (438) | 19.6 (332) | 14.2 (106) | 18.3 (438) | 32.1 (1784) | 20.9 (416) | 30.0 (2200) |
| Songwa/Mbeya | 54.1 (311) | 46.5 (99) | 52.2 (410) | 28.0 (311) | 29.4 (99) | 28.3 (410) | 41.5 (1371) | 33.1 (335) | 39.9 (1706) |
| Tabora | 92.1 (329) | 85.5 (97) | 90.6 (426) | 49.2 (329) | 57.7 (97) | 51.2 (426) | 73.8 (2395) | 76.7 (433) | 74.2 (2828) |
| Tanga | 54.4 (364) | 30.6 (62) | 50.9 (426) | 26.9 (364) | 12.9 (62) | 24.9 (426) | 37.2 (1724) | 20.4 (230) | 35.2 (1954) |
| Zanzibar North | 82.2 (298) | 82.8 (29) | 82.3 (327) | 45.9 (298) | 45.0 (29) | 45.9 (327) | 67.0 (1625) | 71.5 (116) | 67.3 (1741) |
| Zanzibar Sth/Cntrl | 83.0 (299) | 76.9 (26) | 82.5 (325) | 50.8 (299) | 61.5 (26) | 51.7 (325) | 67.9 (1528) | 63.2 (76) | 67.6 (1604) |
| Zanzibar West | 63.0 (449) | 33.1 (6) | 62.6 (455) | 25.2 (449) | 33.1 (6) | 25.3 (455) | 45.4 (2488) | 28.5 (7) | 45.4 (2495) |

N indicates the total number of households or household population in each category.

*Geita, Shinyanga, Mwanza, Kagera, and Simiyu were grouped into a single entity (Lake Zone).
