# Supplementary material for: Assessing trends in non-coverage bias in mobile phone surveys for estimating insecticide-treated net coverage: a cross-sectional analysis in Tanzania, 2007–2017
Source: BMJ Public Health. 2025 Mar 4;3(1):e001379. doi: 10.1136/bmjph-2024-001379 (PMC11883883; doi:10.1136/bmjph-2024-001379)
Supplement: online supplemental table 5 [file bmjph-3-1-s007.pdf]

Supplemental Table 5. TZ MIS 2017. Households or household population by RBM-MERG ITN indicator, region, and mobile phone ownership status. Point estimates from bootstrapping method of resampling.

Region	Households with at least one ITN			Households with at least one ITN for every two people			Population with access to an ITN in their household		
	Households with mobile phones % (N)	Households without mobile phones % (N)	All households % (N)	Households with mobile phones % (N)	Households without mobile phones % (N)	All households % (N)	Population in households with mobile phones % (N)	Population in households without mobile phones % (N)	Population among households % (N)
**National**	79.9 (7575)	66.8 (1755)	77.5 (9330)	44.0 (7575)	39.2 (1755)	43.1 (9330)	62.6 (39438)	51.9 (6973)	61.0 (46411)
Arusha	69.0 (187)	43.8 (16)	67.0 (203)	44.9 (187)	37.5 (16)	44.3 (203)	60.6 (774)	40.8 (49)	59.4 (823)
Dar es Salaam	78.3 (291)	66.5 (12)	77.9 (303)	54.3 (291)	50.1 (12)	54.1 (303)	69.8 (1085)	55.3 (29)	69.4 (1114)
Dodoma	82.7 (150)	68.4 (57)	78.8 (207)	45.4 (150)	40.3 (57)	44.0 (207)	63.7 (779)	53.5 (230)	61.4 (1009)
Katavi/Rukwa	65.5 (499)	57.2 (133)	63.7 (632)	25.8 (499)	31.6 (133)	27.1 (632)	42.7 (2998)	40.5 (547)	42.3 (3545)
Kigoma	78.7 (343)	67.8 (81)	76.6 (424)	35.0 (343)	29.5 (81)	33.9 (424)	59.2 (1913)	47.5 (354)	57.3 (2267)
Kilimanjaro	82.4 (193)	64.9 (20)	80.8 (213)	63.2 (193)	54.9 (20)	62.4 (213)	74.1 (792)	50.0 (42)	72.9 (834)
Lake zone*	81.1 (1699)	67.3 (425)	78.3 (2124)	36.6 (1699)	30.4 (425)	35.4 (2124)	59.7 (10331)	46.7 (2049)	57.6 (12380)
Lindi	86.6 (343)	56.7 (90)	80.4 (433)	60.4 (343)	45.6 (90)	57.3 (433)	75.0 (1370)	56.8 (250)	72.2 (1620)
Manyara	80.3 (158)	57.5 (47)	75.1 (205)	36.0 (158)	25.5 (47)	33.6 (205)	59.8 (899)	44.7 (197)	57.1 (1096)
Mara	89.7 (361)	79.7 (69)	88.1 (430)	47.6 (361)	37.7 (69)	46.0 (430)	73.4 (2094)	58.7 (269)	71.8 (2363)
Morogoro	88.6 (325)	89.0 (91)	88.7 (416)	56.6 (325)	64.8 (91)	58.4 (416)	74.9 (1478)	75.5 (322)	75.0 (1800)
Mtwara	83.3 (323)	65.4 (104)	78.9 (427)	59.8 (323)	50.0 (104)	57.4 (427)	78.4 (1276)	64.4 (292)	75.8 (1568)
Njombe/Iringa	67.4 (301)	56.9 (121)	64.5 (422)	44.2 (301)	36.3 (121)	42.0 (422)	58.6 (1258)	46.8 (395)	55.8 (1653)
Pemba North	81.2 (128)	59.0 (22)	78.0 (150)	36.7 (128)	22.7 (22)	34.6 (150)	61.8 (811)	45.8 (120)	59.7 (931)
Pemba South	89.5 (143)	89.9 (10)	89.5 (153)	52.5 (143)	69.8 (10)	53.6 (153)	78.7 (834)	64.6 (34)	78.1 (868)
Pwani	90.0 (371)	79.5 (44)	89.0 (415)	60.3 (371)	52.3 (44)	59.5 (415)	77.4 (1663)	59.9 (147)	76.0 (1810)
Ruvuma	80.6 (324)	65.5 (96)	77.2 (420)	52.8 (324)	44.8 (96)	51.0 (420)	72.0 (1395)	57.3 (379)	68.9 (1774)
Singida	70.9 (158)	51.1 (47)	66.3 (205)	24.6 (158)	21.3 (47)	23.9 (205)	43.3 (969)	36.7 (210)	42.1 (1179)
Songwa/Mbeya	74.5 (321)	74.9 (100)	74.6 (421)	42.7 (321)	54.9 (100)	45.6 (421)	63.5 (1375)	63.0 (346)	63.4 (1721)
Tabora	70.7 (324)	58.2 (91)	68.0 (415)	27.5 (324)	27.5 (91)	27.5 (415)	45.9 (2152)	49.6 (442)	46.5 (2594)
Tanga	88.0 (182)	91.9 (37)	88.6 (219)	51.7 (182)	59.6 (37)	53.0 (219)	73.0 (828)	79.6 (133)	74.0 (961)
Zanzibar North	84.7 (143)	77.6 (27)	83.5 (170)	53.2 (143)	55.4 (27)	53.5 (170)	69.6 (750)	64.2 (84)	69.1 (834)
Zanzibar Sth/Cntrl	89.8 (137)	67.0 (12)	87.9 (149)	59.9 (137)	58.5 (12)	59.7 (149)	79.2 (583)	56.5 (39)	77.8 (622)
Zanzibar West	70.2 (171)	100.0 (3)	70.7 (174)	29.2 (171)	33.7 (3)	29.3 (174)	49.8 (1031)	85.8 (14)	50.3 (1045)

N indicates the total number of households or household population in each category.

*Geita, Shinyanga, Mwanza, Kagera, and Simiyu were grouped into a single entity (Lake Zone).
